# Prolific S-layer shedding and associated proteins from the methanotroph *Methylomicrobium album* BG8

**DOI:** 10.1128/aem.00607-26

**Published:** 2026-05-29

**Authors:** Mariah K. Hermary, Maria C. Rodriguez Gallo, Rachael Rieberger, Aurelija M. Grigonyte, Kieran McDonald, R. Glen Uhrig, Dominic Sauvageau, Lisa Y. Stein

**Affiliations:** 1 Department of Biological Sciences, University of Alberta 3158, Edmonton, Canada; 2 Department of Chemical and Materials Engineering, University of Alberta 3158, Edmonton, Canada; Georgia Institute of Technology, Atlanta, Georgia, USA

**Keywords:** methanotroph, S-layer, *Methylomicrobium album*, proteome, low pH adaptation, type 1 secretion system

## Abstract

Some methanotrophs synthesize S-layers that overlay their outer membrane. Transmission electron microscopy imaging revealed that *Methylomicrobium album* BG8 constitutively sheds abundant S-layer units, a phenotype not found in seven other methanotrophs, even though *Methylotuvimicrobium buryatense* 5GB1 produces a similar structure. The release of S-layer units occurred regardless of carbon (methane or methanol) or nitrogen (ammonium or nitrate) source, with 50× trace metals, under copper deprivation, and at all growth phases. The released S-layer units were isolated from the culture medium of *M. album* BG8 by density gradient centrifugation for proteome analysis. The proteome revealed S-layer protein subunits, transporters for calcium uptake, including TolC and repeats-in-toxin (RTX) proteins, transporters for uptake of cobalamin and siderophores, cell wall biogenesis proteins, and proteins with Type I secretion system (T1SS) target domains. *M. album* BG8 adapted to grow at pH 4 lost its S-layer, and genome analysis revealed a frameshift mutation resulting in reduced expression of the S-layer unit gene, and an in-frame deletion mutation resulting in almost no expression of an S-layer-associated porin gene. Together, the results suggest that the biogenesis and secretion of *M. album* BG8 S-layer is mediated by its associated T1SS, the S-layer possesses metal acquisition functions, and low pH adaptation of *M. album* BG8 results in the loss of its S-layer, likely due to reduced or incomplete expression of S-layer units and the loss of an associated porin. The involvement of the T1SS and shedding phenotype of the S-layer in *M. album* BG8 could be applied toward selective secretion of proteins and other factors of bioindustrial interest.

**IMPORTANCE:**

The methanotrophic bacterium *Methylomicrobium album* BG8 produces and sheds large quantities of S-layer units into the culture medium regardless of carbon or nitrogen source, metal availability, or growth phase. Of the eight methanotrophic bacteria screened, only *M. album* BG8 possessed this phenotype. Proteomics analysis of density gradient-purified culture supernatant identified the S-layer protein units and proteins involved in metal uptake and S-layer biogenesis, some with secretion signals for the T1SS. *M. album* BG8 adapted to grow at low pH lost the production of its S-layer due to mutations in the genes encoding S-layer units and an associated porin. Better understanding of *M. album* BG8 S-layer production and its shedding phenotype could be harnessed for exporting expressed proteins and bioproducts of industrial interest for the ease of collection and downstream processing.

## INTRODUCTION

Aerobic methanotrophic bacteria are clustered within the Gammaproteobacteria, Alphaproteobacteria, and Verrucomicrobia phyla and are metabolically differentiated by their discrete pathways of carbon assimilation (1). Collectively, methanotrophs are of industrial interest for their capacity to convert methane, a potent greenhouse gas, into valuable molecules, from biopolymers to proteins to biofuels (2, 3). The gammaproteobacterium *Methylomicrobium album* BG8 is a high-priority strain for industrialization due to its metabolic flexibility for rapid growth on methane or methanol, with nitrate or ammonium as its nitrogen source (4, 5), and the availability of a well-curated genome-scale metabolic model (6). Development of methanotrophs as industrial chassis relies on their ability to express materials of commercial interest at scale, requiring the optimization of biomass production and possibly engineering strains to secrete valuable metabolites for the ease of downstream processing (3). The diversity of substrates secreted by Type 1 secretion systems (T1SS) makes this system attractive for the production and extracellular collection of heterologous proteins, biologics (e.g., growth factors), biosensors, and other molecules (7). Methanotrophs with an active T1SS could potentially be engineered to consume methane for the synthesis and secretion of valuable products that promote biodegradation, metal-scavenging, and plant growth, thus enabling simultaneous greenhouse gas removal and its valorization.

Structural analysis of methanotroph cell surfaces has revealed that some strains possess unique S-layers comprised of tightly packed cup-shaped protein units with p6 hexagonal symmetry (8–11). It was hypothesized that these structures could provide rigidity to the cell wall and play a role in osmoregulation and tolerance to extremes of salinity and alkalinity (11). A recent study of S-layers from two alkaliphilic *Methylotuvimicrobium* strains validated this hypothesis (12). However, non-halotolerant and neutralophilic methanotrophs, such as *M. album* BG8 (13) and *Methylomicrobium* sp. HG-1 (14), express similar arrays of these cup-like structures on their surfaces, which brings their physiological role and mechanism of biogenesis into question. *Caulobacter crescentus* forms similarly structured S-layers from cup-shaped RsaA proteins with p6 symmetry (15). These RsaA proteins are secreted through a T1SS and contain a span of six repeat-in-toxin (RTX) domains that bind calcium to enable proper folding and stabilization of the crystalline S-layer lattice on the outer membrane (16). Mutations in the N-terminus membrane attachment site of the RsaA protein result in a prolific shedding phenotype (17). Comparison of the *M. album* BG8 RsaA homolog to that of the methanotrophs *Methylotuvimicrobium buryatense* 5GB1 and *Methylotuvimicrobium alcaliphilum* 20Z showed only ca. 20% sequence identity (12). However, all three methanotroph genomes possess a complete T1SS operon along with their RsaA gene, suggesting that S-layer biogenesis relies on the T1SS, similar to that of *C. crescentus*.

The present study shows that *M. album* BG8 constitutively sheds its cup-shaped S-layer units under a variety of nutrient conditions. The S-layer units from the supernatant of *M. album* BG8 cultures were purified using density gradient centrifugation, and proteomic analysis by LC-MS/MS was performed. *M. album* BG8 was adapted to grow at acidic pH, which caused the loss of its S-layer altogether. This study revealed an unusual capacity for *M. album* BG8 to release large quantities of its S-layer units. S-layer production via a Type I secretion system, calcium-dependent assembly, and a pH-sensitive loss of S-layer collectively underlie the findings. Understanding and repurposing S-layer production and its shedding could enable next-generation biomanufacturing platforms to convert abundant single-carbon feedsotcks into valuable, easily recoverable, proteins.

## RESULTS

### *M. album* BG8 constitutively releases abundant cup-shaped S-layer units

**Fig 1:**
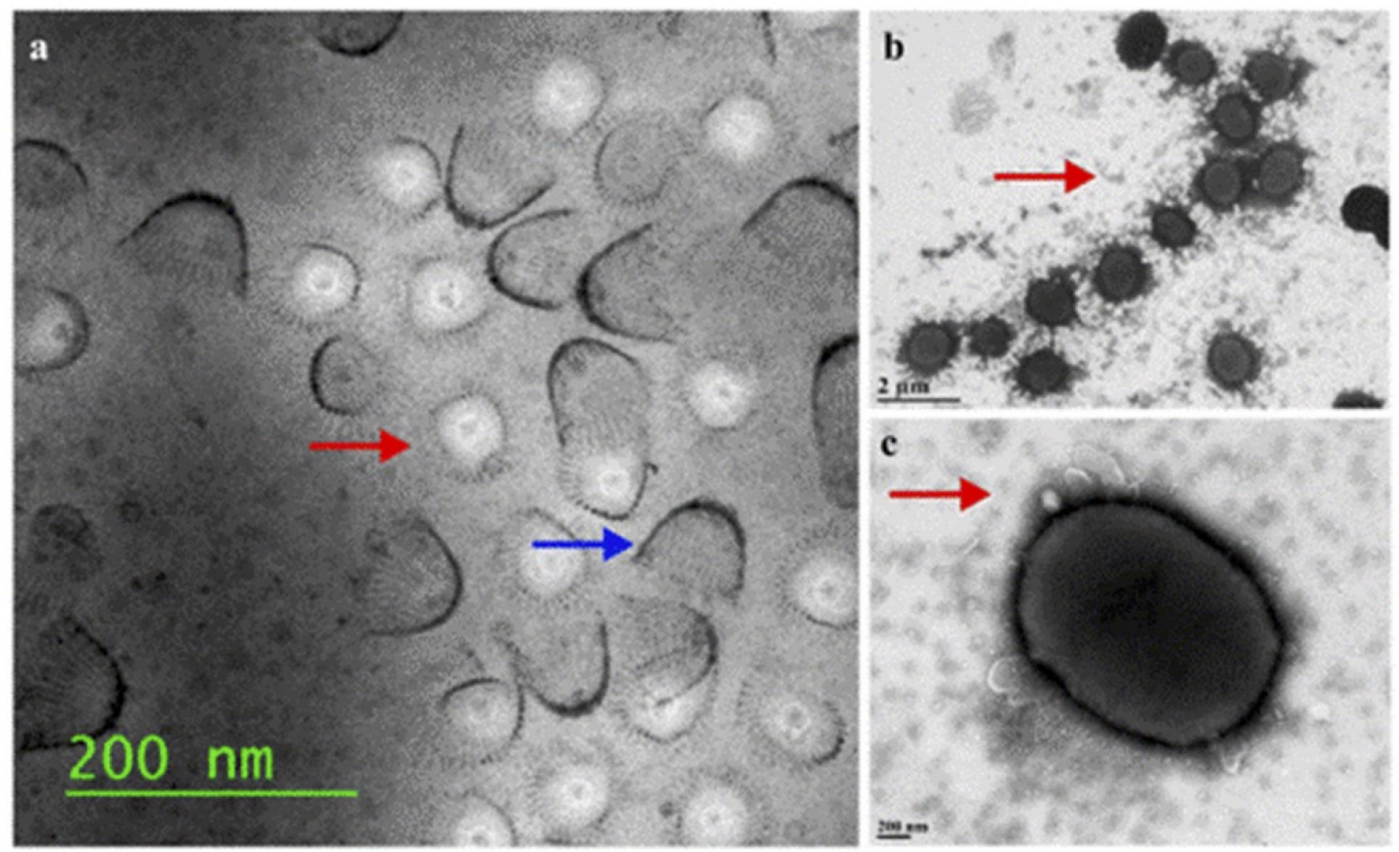
Transmission electron micrographs of *M. album* BG8 with released S-layer protein units (as depicted with arrows) when cultured with methane in various media conditions. (**a**) NMS medium, (**b**) AMS medium without copper, and (**c**) NMS medium with 50× trace elements solution. Micrographs contain a wide range of scales to highlight membrane morphology and adjacent surrounding areas.

**Fig 2:**
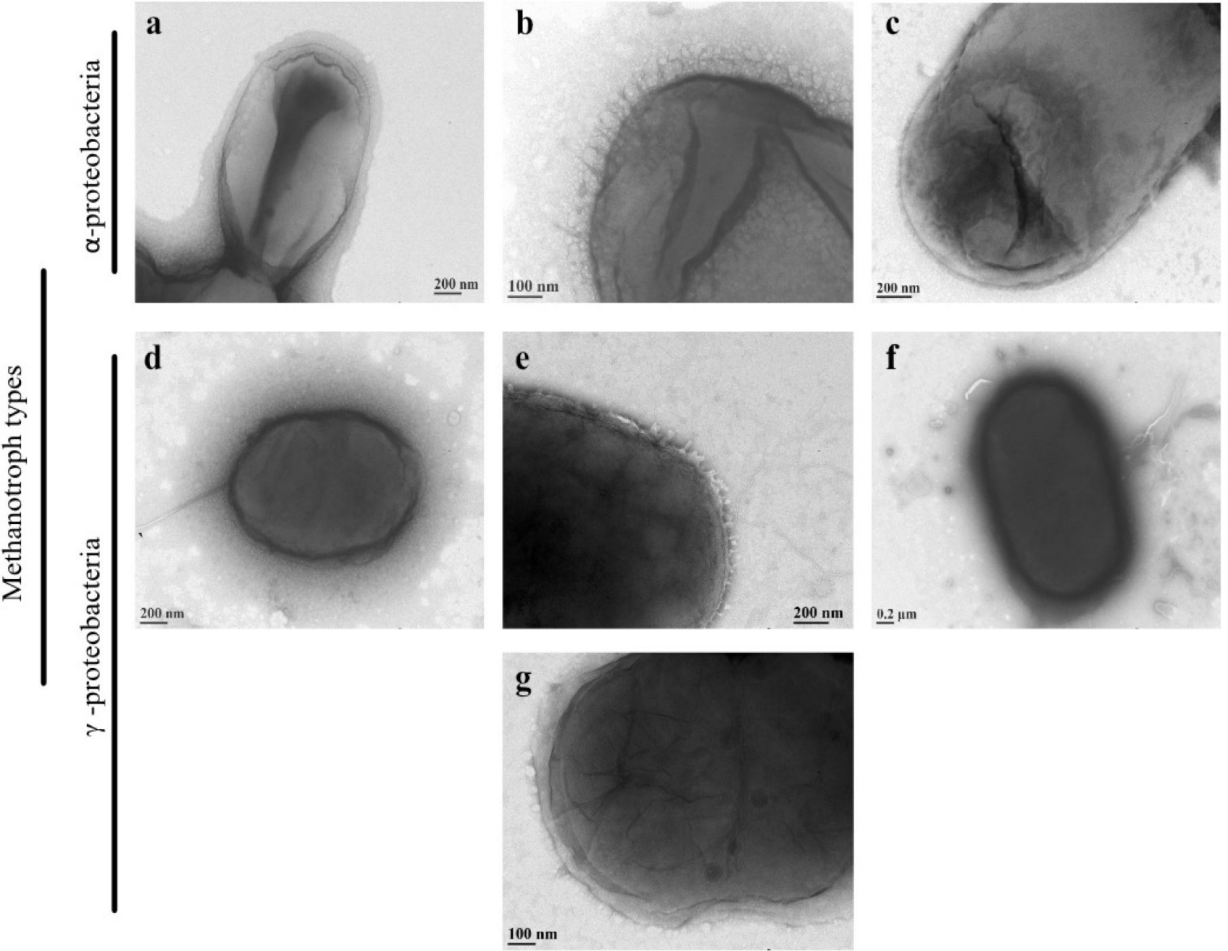
Transmission electron micrographs of selected alpha- and gamma-proteobacterial methanotrophs cultivated on methane and NMS. (**a**) *Methylocystis* sp. WRRC1, (**b**) *Methylocystis* sp. Rockwell, (**c**) *Methylosinus trichosporium* OB3b, (**d**) *Methylomonas denitrificans* FJG1, (**e**) *Methylicorpusculum oleiharenae* XLMV4^T^, (**f**) *Methylotuvimicrobium buryatense* 5GB1, and (**g**) *Methylococcus capsulatus* Bath.

Transmission electron microscopy [TEM] of *M. album* BG8 displayed cup-shaped particles (Fig. 1), with diameters ranging from 49.5 to 75.2 nm, with an average of 60.9 ± 5.7 nm (*n* = 50; Table S1). Released S-layer units were observed when *M. album* BG8 was grown in nitrate (NMS) or ammonium mineral salts (AMS) media containing methane or methanol as a sole carbon source, media devoid of copper, and media containing 50× trace elements solution (examples of TEM images for various conditions are shown in Fig. 1). Thus, the release of S-layer protein units was considered constitutive, as it did not vary with changes in carbon source, nitrogen source, or metal availability. Released S-layer protein units were also present immediately after culture inoculation, in mid-log growth, and at the stationary phase (images not shown). However, released S-layer protein units were not detected in the medium of seven other methanotrophic strains examined (Fig. 2), including *Methylotuvimicrobium buryatense* 5GB1, which possess similar cup-shaped S-layer lattices on its cell surface (11).

### S-layer unit proteome suggests associated functions of T1SS, calcium uptake, metal acquisition, and membrane assembly

**Fig 3:**
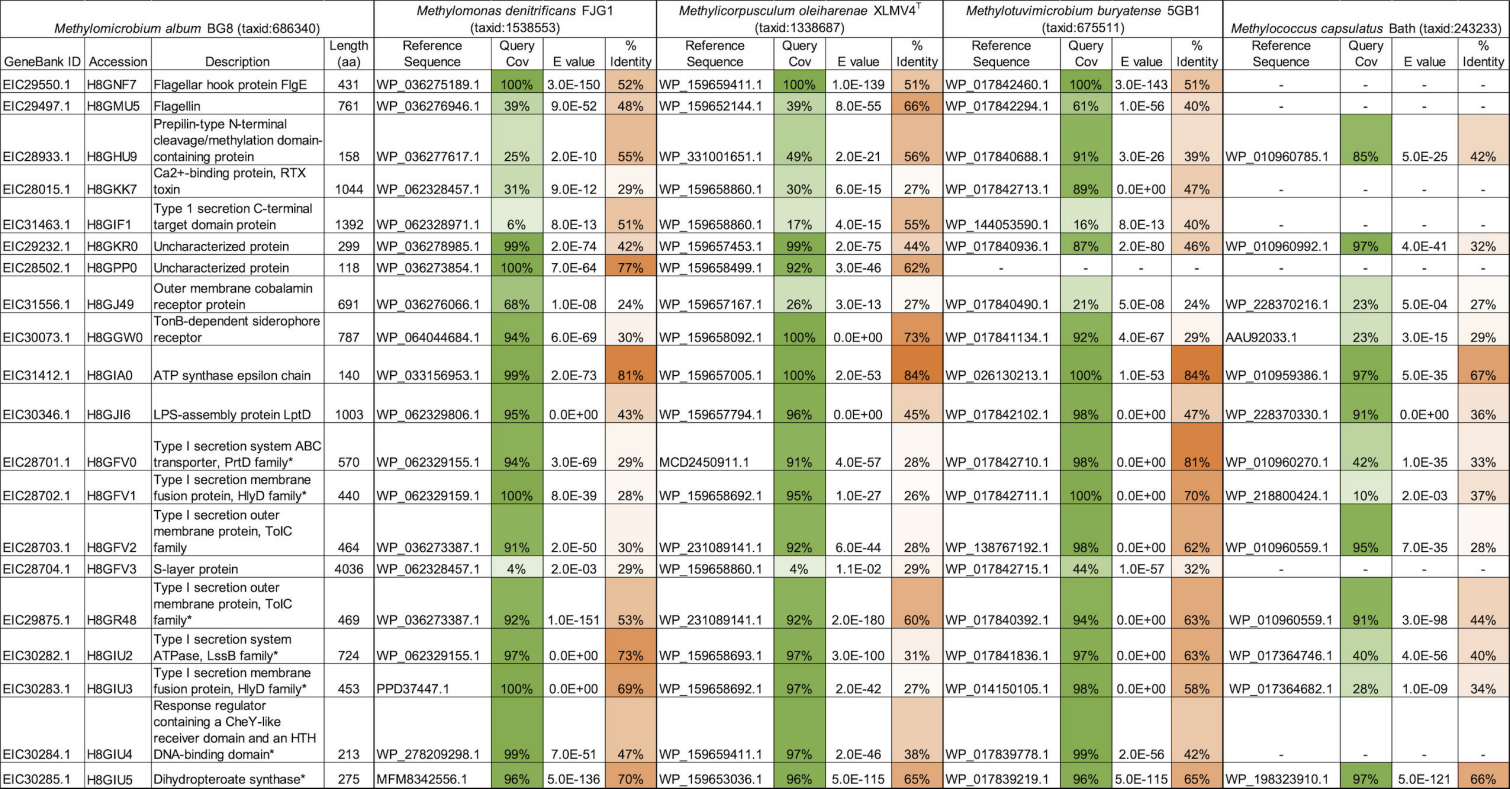
Protein sequence comparison of S-layer and T1SS-related proteins of *M. album* BG8 to gammaproteobacterial methanotrophs *M. denitrificans* FJG1, *M. oleiharenae* XLMV4^T^, *M. buryatense* 5GB1, and *M. capsulatus* Bath.

From the total of 310 peptides identified by LC-MS/MS from purified S-layer units released into the medium of *M. album* BG8 cultures (Table S2), 14 had high statistical significance across triplicate samples (ranking Peptide Spectrum Match [PSM]> 4, peptides > 5, and score >20). Of note were the S-layer protein itself (H8GFV3), proteins related to binding and uptake of calcium and S-layer assembly (Ca^2+^-binding RTX protein, PtrC metalloprotease, and pre-pilin N-terminal cleavage) (16, 18, 19), proteins for siderophore and divalent cation uptake (TonB receptor) and cobalamin transport (OM receptor), a porin protein (H8GGQ6), a protein with a T1SS C-terminal target domain (H8GIF1), and a LPS-assembly protein (Table 1, Fig. 3).

**TABLE 1:** Significant peptide hits from LC-MS/MS detection of the purified released S-layer protein units (*n* = 3), organized by Genbank locus tag^*b*^

Genbank locus tag	Uniprot accession	UniProt description	Mean PSM	Mean peptide	Mean score	COG category
Metal_0147	H8GKK7	Ca2+-binding protein, RTX toxin	5.7	5.7	18.5	Secondary metabolites biosynthesis, transport, and catabolism
Metal_0479	H8GNA1	Uncharacterized protein (Metallo-protease PrtC^*a*^)	30.3	15.7	106.7	Secondary metabolites biosynthesis, transport, and catabolism
Metal_0659	H8GPP0	Uncharacterized protein	7	1	20	Inorganic ion transport and metabolism
**Metal_0880^*c*^**	**H8GFV3**	**S-layer protein**	**7.3**	**6.3**	**23.2**	**Secondary metabolites biosynthesis, transport, and catabolism**
Metal_1116	H8GHU9	Prepilin-type N-terminal cleavage/methylation domain-containing protein	64.3	4	231.4	Intracellular trafficking, secretion, and vesicular transport
Metal_1447	H8GKR0	Outer membrane protein beta-barrel domain-containing protein^*a*^	5	2	17.9	Cell wall/membrane/envelope biogenesis
Metal_1728	H8GMU5	Flagellin	331.7	22.3	1316.7	Cell motility
Metal_1783	H8GNF7	Flagellar hook protein FlgE	16	8.7	51.7	Cell motility
Metal_2279	H8GGQ6	Uncharacterized porin	16	4.7	52.5	Cell wall/membrane/envelope biogenesis
Metal_2337	H8GGW0	TonB-dependent siderophore receptor	7	6.3	20.2	Inorganic ion transport and metabolism
Metal_2634	H8GJI6	LPS-assembly protein LptD	16.7	2.7	43.8	Cell wall/membrane/envelope biogenesis
Metal_3768	H8GIA0	ATP synthase epsilon chain	9.7	1	22.4	Lipid transport and metabolism
Metal_3821	H8GIF1	Type I secretion C-terminal target domain protein	24.5^+^	12.5^+^	71.9^+^	Secondary metabolites biosynthesis, transport, and catabolism
Metal_3919	H8GJ49	Outer membrane cobalamin receptor protein	8	6.3	22.5	Coenzyme transport and metabolism

^*a*^ *HHpred protein annotation (>95% probability).

^*b*^ "+" replicates *n* = 2.

^*c*^ Bolded protein, major S-layer unit.

Proteins with relevance to S-layer biogenesis, but with lower statistical significance (PSM <5, peptides < 5, score <20), included TolC, which is the outer-membrane channel component of the T1SS that interacts with the Ca^2+^ binding domains of RTX proteins for their secretion (Table 2) (20). Several membrane-related proteins with lower statistical significance included a cation diffusion facilitator protein, a general secretion pathway “protein D,” and TolB from the Tol-Pal system. OmpA protein, also associated with the S-layer proteins, may play a role in S-layer biogenesis or tethering to the cell wall, as it is an abundant and vital protein in the outer membrane of gram-negative bacteria (21). CorA, a protein once thought to anchor S-layer proteins to the cell wall (10), was only found in one of the triplicate proteome samples at low peptide counts. High counts of flagella-related proteins were also co-purified from the culture supernatant.

**TABLE 2:** Additional protein hits of interest from LC-MS/MS detection of purified released S-layer protein units organized by Genbank locus tag^*a*^

Genbank locus tag	UniProt Accession	UniProt description	Mean PSM	Mean peptide	Mean score	COG category
Metal_0094	H8GK04	Cation diffusion facilitator family transporter	2.5	1.00^*c*^	6.19	Inorganic ion transport and metabolism
Metal_0482	H8GNA4	Uncharacterized protein	4.67	2	11.77	–^*e*^
Metal_0879	H8GFV2	Type I secretion outer membrane protein, TolC family	3	3	8.09	Cell wall/membrane/envelope biogenesis; Intracellular trafficking, secretion, vesicular transport.
Metal_1031	H8GH59	PAS domain S-box/diguanylate cyclase (GGDEF) domain-containing protein	3.50^*b*^	1	9.16	Signal transduction mechanisms
Metal_1419	H8GK80	Outer membrane protein/peptidoglycan-associated (Lipo)protein	3	2.33	8.32	Cell wall/membrane/envelope biogenesis
Metal_1666	H8GM80	Autotransporter secretion inner membrane protein TamB	3.33	1	7.05	–
Metal_1730	H8GMU7	Flagellar hook-associated protein 2	5	5	13.49	Cell motility
Metal_1789	H8GNG3	Flagellar hook-associated protein 1	2.67	2.67	8.48	Cell motility
Metal_1790	H8GNG4	Flagellar hook-associated protein 3	2.33	2.33	6.55	Cell motility
Metal_1986	H8GPX1	Citrate lyase beta subunit	2.33	1	8.01^*d*^	Carbohydrate transport and metabolism
Metal_1998	H8GPY3	Outer membrane protein/peptidoglycan-associated (Lipo)protein	2.67	2.33	7.49	Cell wall/membrane/envelope biogenesis
Metal_2207	H8GG32	Adenosylhomocysteinase	3.33	1.33	4.8	Coenzyme transport and metabolism
Metal_2282	H8GGQ9	DNA-binding transcriptional regulator NtrC	2.67	1	6.79	Signal transduction mechanisms
Metal_2345	H8GGW4	Uncharacterized protein	2.67	1	6.78	Function unknown
Metal_2446	H8GI24	Uncharacterized protein	4.00^*b*^	1	10.57	–
Metal_2469	H8GI44	PQQ-dependent dehydrogenase, methanol/ethanol family	3	3	7.4	Carbohydrate transport and metabolism
Metal_2481	H8GI56	Uncharacterized protein	2.50^*b*^	2.5	6.29	–
Metal_2515	H8GI88	Probable cytosol aminopeptidase	2.33	2	6.21	Amino acid transport and metabolism
Metal_2819	H8GL29	Tol-Pal system protein TolB	4.67	4.33	14.22	Intracellular trafficking, secretion, and vesicular transport
Metal_2821	H8GL31	Cell division coordinator CpoB	5	5	11.65	Cell cycle control, cell division, chromosome partitioning
Metal_3055	H8GN21	Uncharacterized protein	4	1	8.92	Function unknown
Metal_3106	H8GNM8	Hemolysin-coregulated protein	3.67	3	10.3	Function unknown
Metal_3255	H8GPH9	Murein lipoprotein	1.67	1.67	6.06	Function unknown
Metal_3338	H8GQ61	Uncharacterized protein	3	2.67	7.27	Function unknown
Metal_3460	H8GRD0	Transaldolase	4.33	4	10.21	Nucleotide transport and metabolism
Metal_3679	H8GHK8	Gly-zipper_OmpA domain-containing protein	3.5	2	12.03^*d*^	Function unknown
Metal_3822	H8GIF2	VCBS repeat-containing protein	2.67	2	11.46^*d*^	Secondary metabolites biosynthesis, transport, and catabolism
Metal_3828	H8GIF8	General secretion pathway protein D	3	2.5	7.94^*d*^	Cell motility; intracellular trafficking, secretion, and vesicular transport

^*a*^ HHpred protein annotation (>95%).

^*b*^ Absent in replicate A.

^*c*^ Absent in replicate B.

^*d*^ Absent in replicate C.

^*e*^ "–" denotes no COG category identified.

Homologs of significantly identified proteins from the proteome were searched for in the genomes of the four gammaproteobacterial methanotrophs screened in this study. Only *M. buryatense* 5GB1 encoded the T1SS gene cluster that includes a homolog of the RsaA S-layer protein (Accession: H8GFV0-H8GFV3); this was expected, as this was the only other methanotroph we included that has an S-layer similar to that of *M. album* BG8 (12) (Fig. 3). Although not expressed in the supernatant proteome, one other gene cluster encoding T1SS ATPase, T1SS HylD membrane fusion protein, a response regulator, and a dihydropteroate synthase (Accession: H8GIU2-H8GIU5) was identified along with a nearby gene for T1SS TolC (Accession: H8GR48). Sequences of these genes in other methanotrophs showed varying percentages of sequence identity to the genes from *M. album* BG8 (Fig. 3), but it is unknown if this second T1SS gene cluster is related to S-layer biogenesis.

### Loss of S-layer in a strain of *M. album* BG8 adapted to grow at low pH

**Fig 4:**
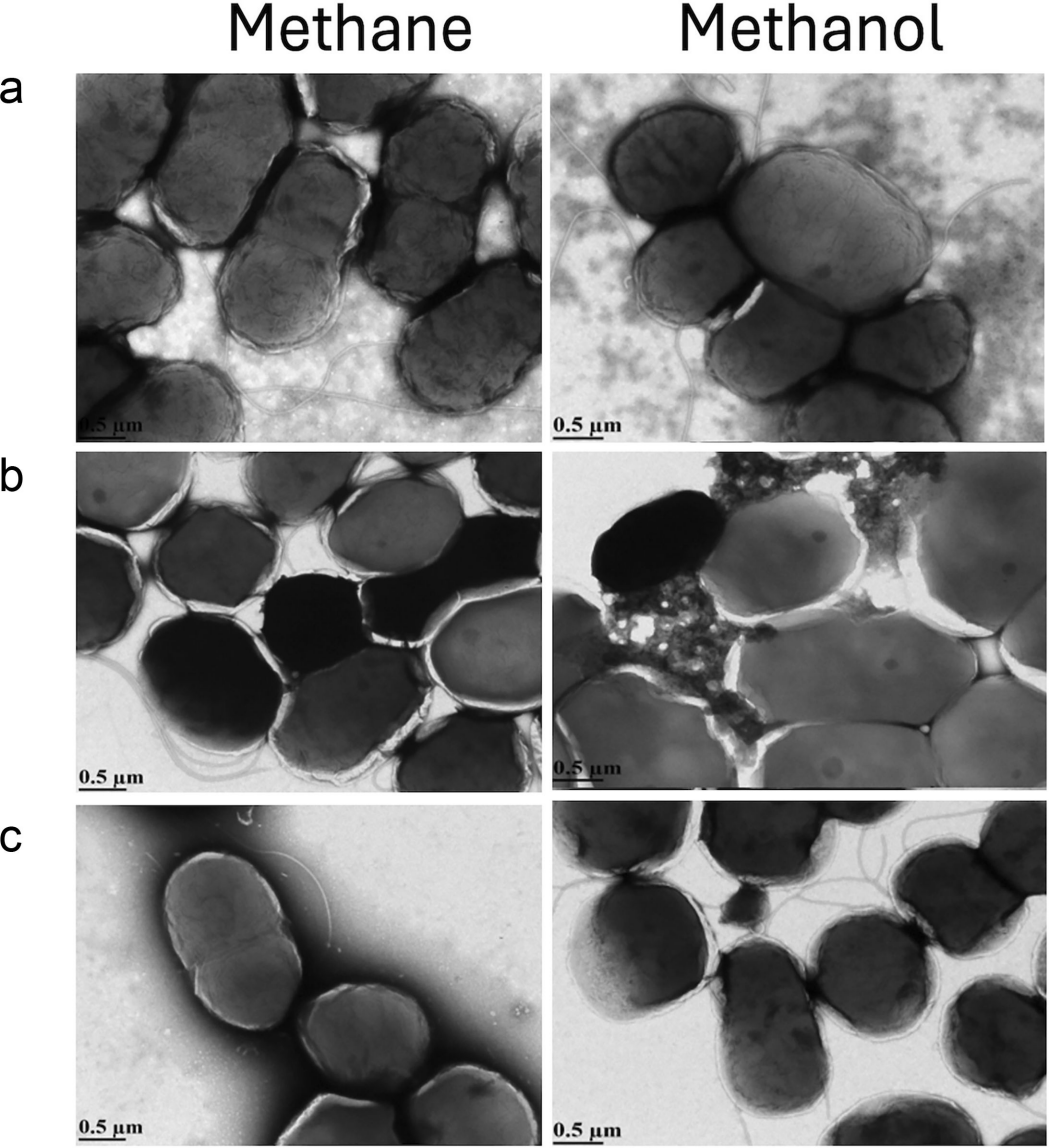
(**a**) *M. album* BG8 (parental strain, unadapted) grown in NMS at pH 6.8 in methane or methanol, showing prolific shedding of S-layer particles and the presence of flagella in the surrounding media. (**b**) *M. album* BG8 adapted to grow at pH 4, cultivated in methane or methanol, showing the absence of S-layer particles in the surrounding media. (**c**) Low pH-adapted *M. album* BG8 grown at pH 6.8 in methane or methanol NMS, showing the continued absence of S-layer particles in the surrounding media.

To test whether growth at acidic pH had a destabilizing effect on S-layer biogenesis and shedding, as has been shown in *Caulobacter crescentus* (17), we conducted adaptive laboratory evolution on *M. album* BG8 to grow in media from pH 6.8 to 4. Successive transfers in acidified NMS medium resulted in three separate lineages of low pH-adapted cultures. TEM images of the adapted cultures grown on methane or methanol showed smooth cell surfaces devoid of S-layer and the absence of shed S-layer particles into their culture medium (Fig. 4). Genome sequences between the low pH-adapted and parental (unadapted) *M. album* BG8 strains were compared. A frameshift mutation in the S-layer-encoding gene (Metal_0880) at nucleotide 2,987/12,111, with a Leu substitution at amino acid 996/4,036, was identified (Data set S1; Table 3). Transcriptomic analysis showed that expression of the Metal_0880 gene and its associated T1SS genes (Metal_0877-0879) (12) was ca. 50% lower in the low pH-adapted than in the parental *M. album* BG8 grown in NMS medium at pH 6.8 (Table 3) (5). A frameshift mutation in the gene for a highly expressed porin protein (H8GGQ6) in the S-layer proteome was also identified (Metal_2279), and its transcription was nearly abolished in the low pH-adapted strain compared to the parental strain (Table 3). These two mutations alone may have resulted in the smooth surface of the low pH-adapted strain due to incomplete S-layer unit expression and stabilization or anchoring of S-layer units to the cell wall by the H8GGQ6 porin protein. Thus, adapting *M. album* BG8 to grow at low pH resulted in mutations relevant to S-layer biogenesis, yielding complete loss of its S-layer (Fig. 4). No difference in growth rate was detected between the low pH-adapted and parental *M. album* BG8 strains when cultivated in NMS medium at pH 6.8 (Fig. S1). Only the low pH-adapted strains could grow in NMS medium at pH 4, albeit at a slower growth rate than at pH 6.8 (Fig. S1). The results suggest that the energetic cost of S-layer production did not affect the growth rate of *M. album* BG8 in neutral pH media and that the low pH adaptation process resulted in loss of its S-layer.

**TABLE 3:** Mutations and level of transcription (transcripts per million) identified in the genome and RNA-seq data of *M. album* BG8 adapted to grow at pH 4^*d*^^,^^*e*^

Genbank locus tag	UniProt accession	Type of mutation	UniProt function	TPM^*a*^ adapted	TPM^*a*^ parental
Metal_0463	H8GN86	Synonymous	Serine/threonine protein kinase	105	61
Metal_0492	H8GNB4	Synonymous	Malto-oligosyltrehalose synthase	28	3
*Metal* *_0877^b^*	*H8GFV0*	*none*	*Type I secretion system ABC transporter, PrtD family4*	*128*	*252*
*Metal_0878^b^*	*H8GFV1*	*none*	*Type I secretion membrane fusion protein, HlyD family*	*97*	*167*
*Metal_0879^b^*	*H8GFV2*	*none*	*Type I secretion outer membrane protein, TolC family*	*81*	*181*
Metal_0880	H8GFV3	Frameshift (delT at nt 2,987; Leu at aa 996	S-layer protein	1764	3,948
Metal_0915	H8GFY7	Insertion	Drug resistance transporter, EmrB/QacA subfamily	46	67
Metal_0961	H8GGI4	Frameshift	Choline dehydrogenase-like flavoprotein	104	20
Metal_1085	H8GHB2	Synonymous	Ketol-acid reductoisomerase (NADP(+))	449	703
Metal_1113	H8GHU6	Glu – Val	Uncharacterized protein	154	44
Metal_1464	H8GKS7	Asp – Gly	DNA-binding protein H-NS	2960	2618
Metal_1726	H8GMU4	Asp – Ser	Flagellar protein FliL	70	22
Metal_1910	H8GP90	Synonymous	Rhodanese-related sulfurtransferase	56	66
Metal_1927	H8GPA7	Val – Ile	Putative PLP-dependent enzyme possibly involved in cell wall biogenesis	115	96
Metal_2019	H8GQF3	Asp – Asn	Succinate dehydrogenase hydrophobic membrane anchor subunit	135	105
Metal_2096	H8GQM4	Synonymous	Putative thymidine phosphorylase	77	76
Metal_2101	H8GR29	Val – Gly	Probable membrane transporter protein	18	8
Metal_2132	H8GR59	Synonymous	Cadherin domain-containing protein	66	35
Metal_2140	H8GR66	Val – Leu	Protein-export membrane protein SecG	767	1324
**Metal_2279**	**H8GGQ6**	**in-frame deletion**	**Porin**	**21**	**865**
Metal_2323	H8GGU6	Glu – Gly	Cytochrome c	45	58
Metal_2426	H8GHJ6	Deletion	RHS repeat-associated core domain protein	20	5
Metal_2567	H8GIU3	Deletion	Membrane fusion protein (MFP) family protein	132	91
Metal_2569	None^*c*^	Insertion	TolC family outer membrane protein	873	428
Metal_2678	H8GJM5	Glu–Ala	Tfp pilus assembly protein PilN	95	48
Metal_2685	H8GJN2	Leu–Pro	C-terminal processing peptidase	215	407
Metal_2779	H8GKZ0	Deletion	Transcriptional regulator	35	40
Metal_3143	H8GNR1	One missense, two synonymous	Transposase	40	40
Metal_3171	H8GNT9	Ser–Asn	Phosphatidylserine decarboxylase proenzyme	72	271
Metal_3777	H8GIA9	Val–Ala	Cobalt-zinc-cadmium resistance protein CzcC	60	53
Metal_3987	H8GJT4	Leu–Pro	Transglutaminase-like domain-containing protein	36	93

^*a*^ TPM: mRNA transcripts per million; data was collected for *M. album *BG8 growing in NMS medium at pH 6.8.

^*b*^ mRNA expression values of the three T1SS genes associated with the S-layer structural gene are reported to compare between the low pH-adapted and parental strains.

^*c*^ UniProt accession number not available; protein function supported by InterProScan and blastp annotation.

^*d*^ The adapted genome was compared to the parental genome. Gene expression levels in the adapted and parental transcriptomes are reported. The two genes in bold were identified in the S-layer proteome.

^*e*^ Italicized genes denote the three T1SS components encoded with the S-layer encoding gene, Metal_0880.

## DISCUSSION

*M. album* BG8 was initially isolated by Roger Whittenbury in 1971 and has been continuously maintained in culture ever since (22). It is likely that the dissociation of its S-layer observed here was acquired by random mutation over the thousands of generations that occurred in laboratory cultures and is not a natural feature of the original isolate. Analysis of dissociated S-layer units offers the opportunity to understand more about the S-layer structure, its biogenesis, and its purpose in *M. album* BG8 and provides a basis for the potential utilization of the shedding phenotype to produce and collect products of industrial interest. Based on the most abundant proteins with annotated functions in the proteome of released *M. album* BG8 S-layer units, its biogenesis appears to involve calcium-mediated secretion of S-layer proteins via Ca^2+^-binding RTX domains of the associated T1SS (19, 20) and assembly with assistance of several outer membrane and other RTX-domain proteins (18, 19), including anchoring to the cell wall by highly expressed porin or other outer membrane proteins (23). The T1SS has three components: an ABC transporter located within the inner membrane, a membrane fusion protein that spans the periplasmic space, and an outer membrane porin protein to create an ATP-dependent translocation mechanism (20). TolC, a highly represented protein in the *M. album* BG8 S-layer unit proteome, forms the outer membrane porin component of the T1SS and transports Ca^2+^-binding proteins via a Ca^2+^ ratcheting mechanism (20). The protein targets of the T1SS are typically defined by several blocks of nonapeptide-binding consensus sequences located at their C-termini (24). The identification of significant RTX exoprotein and T1SS C-terminal target domain protein in the S-layer unit proteome suggests that these T1SS-secreted proteins are associated with the S-layer structure. The significant presence of associated PrtC metalloprotease suggests a putative role in S-layer biogenesis or maintenance, analogous to what is observed with the Sap metalloprotease of *C. crescentus* (17).

The S-layer protein (H8GFV3) of *M. album* BG8 is 4,036 amino acids long and contains a conserved hemolysin-type (RTX) calcium-binding region (IPR018511) (19) and a CalX-like domain (IPR038081), with only 35% amino acid identity to the S-layer protein identified in *M. buryatense* 5GB1 (Fig. 3). S-layer proteins are known to display broad diversity between species (25), and the amino acid identity observed between the sequences of S-layer proteins from *M. album* BG8 and *M. buryatense* 5GB1 is above the typical threshold required to consider shared functions (26). In addition, similar to what is found in *M. album* BG8, a Ca^+^-binding domain was identified in the RsaA S-layer protein of *C. crescentus* (15). Other Gammaproteobacteria that encode homologs to the H8GFV3 S-layer protein of *M. album* BG8 include the close relative *Methylomicrobium lacus* (49% AA ID) and several strains of *Marinobacterium georgiense* (36%–37% AA ID) (Table S4), for which S-layers and membrane blebs have been reported (27). The S-layer proteins of *M. album* BG8, *M. buryatense* 5GB1, and *M. alcaliphilum* 20Z are encoded in gene clusters along with cognate T1SS proteins (H8GF0, H8GF1, and H8GF2) (28). Here, we showed congruent expression of the three T1SS and S-layer protein genes in both the low pH-adapted and parental strains of *M. album* BG8 through transcriptomic analysis (Table 3), suggesting the co-regulation of this gene cluster. Exposure of *C. crescentus* to low pH causes release of its S-layer, which has been used to collect S-layer particles for deeper analysis (17). For *M. album* BG8, the S-layer was already actively dissociating from the cell at neutral pH, and further adaptation to low pH resulted in the complete loss of S-layer, likely due to a frameshift mutation in the S-layer-encoding gene and an in-frame deletion in an S-layer-associated porin gene (Table 3).

The copper-regulated CorA and CorB proteins of *M. album* BG8 possess Ca^2+^-binding channels (8, 29), and in conjunction with the TonB-ExbB-ExbD transporter system, they can mediate copper and divalent cation uptake and ion homeostasis (8). Correspondingly, two of the proteins with high counts in the S-layer unit proteome were the TonB siderophore receptor protein (H8GGW0) and a Ca^2+^-binding protein (H8GKK7). However, CorA was only found with low counts in one proteome replicate (Table S2). Although CorA was once considered a possible anchor point for S-layer proteins (10), a recent study on the S-layer of *M. buryatense* 5GB1 showed that its deletion did not affect S-layer production or attachment but did affect copper uptake (12). As neither copper starvation nor growth in 50× trace element solution changed the pattern of S-layer production and release in *M. album* BG8, the shedding phenotype does not appear to be regulated by the abundance of trace metals. Alternatively, the highly expressed porin protein (H8GGQ6) that contained a deletion in the low pH-adapted *M. album* BG8 or the outer membrane beta-barrel domain protein (H8GKR0) could be investigated as potential cell wall anchors, similar to the porin-like proteins of *Deinococcus radiodurans* (23). It is also possible that the LPS assembly protein (H8GJI6) could be a linkage of S-layer to the cell wall in a calcium-dependent fashion similar to that of *C. crescentus* (30).

Although S-layers can be produced by various methanotrophs, *M. album* BG8 was the only one out of eight examined from both Alpha- and Gammaproteobacteria to prolifically release S-layer units into the culture. Structures in TEM images surrounding methanotrophs other than *M. album* BG8 included white patches from the phosphotungstic acid-negative stain (Fig. 2), membrane protrusions around the surface of *M. oleiharenae* XLMV4^T^ cells (Fig. 2e) (31), sparse spherical particles around some *M. buryatense* 5GB1 cells (Fig. 2f), and a capsule layer surrounding *M. capsulatus* Bath (Fig. 2g) (22). None of these structures resembled the released S-layer proteins of *M. album* BG8, but the patchy occurrence of S-layers and capsules among the methanotrophic bacteria continues to be an intriguing topic for future study.

### Application of the T1SS and released S-layer proteins for bioindustrial protein production

The industrialization of methanotrophs has been growing in appeal due to the potential to convert methane, a potent greenhouse gas and cheap feedstock, into value-added products such as small metabolites, biofuel precursors, biopolymers, and proteins (32). The phenotype of *M. album* BG8, wherein its S-layer units are delivered from the cytoplasm to the cell surface by the T1SS (33) and then released to the external environment, represents an advantageous protein production platform. Using the shedding S-layer system, different strategies can be targeted: a heterologous protein can be fused to residues used for excretion through the T1SS for expression of single protein molecules, or the protein of interest can be fused to the N-terminal of the S-layer protein (34) to produce functionalized S-layer units within the culture medium. Of course, the success of such strategies is dependent on the structure and function of the protein of interest, but in each case, the recovery of a heterologous product may be facilitated by the excretion and release mechanism of S-layer units, greatly simplifying downstream processing and reducing associated costs.

### Conclusion

This study identified and characterized the S-layer shedding phenotype of *M. album* BG8 that is not shared by seven other screened methanotrophic strains. The prolific production and release of *M. album* BG8 S-layer units into the culture medium—likely a consequence of long-term cultivation of this strain—allowed for their purification and proteomic analysis. We identified co-expression of the S-layer protein gene with its neighboring T1SS genes, and in the proteome, we observed the S-layer units, TolC proteins, proteins with T1SS signal sequences, metal-acquisition and membrane biogenesis proteins, and a prominent porin. *M. album* BG8 adapted to grow at low pH through adaptive laboratory evolution-acquired mutations in genes encoding the S-layer protein and the prominent porin, resulting in low or abolished expression, respectively. These two mutations may be responsible for the constitutive loss of the S-layer in the low pH-adapted strain. In *C. crescentus*, S-layer proteins comprise up to 30% of the total cellular protein, and considerable energy is required to generate and release S-layer proteins. However, *M. album* BG8 grows efficiently and rapidly on both methane and methanol, with a relatively high growth rate compared to other methanotrophs despite its constitutive production and shedding of S-layer (4). The S-layer production and shedding phenotype of *M. album* BG8 presents an opportunity to produce and secrete bioproducts of industrial interest, including heterologous proteins, growth factors, and metal-chelating agents, among others. Although the cellular energy demands for constitutive S-layer production are high, an added benefit of using *M. album* BG8 is that increased methane consumption will foster optimized rates and yields of bioproduction, which can also yield a larger net climate benefit.

## MATERIALS AND METHODS

### Culturing conditions and media

Eight methanotroph strains maintained in the laboratory were used: Gammaproteobacteria (*Methylomicrobium album* BG8, *Methylomonas denitrificans* FJG1, *Methylicorpusculum oleiharenae* XLMV4T, *Methylococcus capsulatus* Bath, and *Methylotuvimicrobium buryatense* 5GB1) and Alphaproteobacteria (*Methylocystis* sp. WRRC1, *Methylocystis* sp. Rockwell, and *Methylosinus trichosporium* OB3b). Methanotrophs were cultivated in 250-mL Wheaton bottles fitted with screw cap lids inlaid with a butyl rubber septum filled with 100 mL of NMS or AMS medium (4) or NMS2 medium (35) for *M. buryatense* 5GB1. Gas headspace (50 mL) was removed from each bottle, after which methane (60 mL) was injected via a syringe fitted with a 0.22-µm filter. For *M. album* BG8 grown on methanol, 100 µL of high-performance liquid chromatography grade methanol (Sigma-Aldrich) was added. Experiments with increased trace metals included a 50× standard trace element solution. Experiments with copper-free media included Chelex 100 (BioRad)-treated media (except the trace-elements solution) added to acid-washed Wheaton bottles; 1 mL (1%) inoculum from a late exponential phase culture that had been passaged at least once under the identical growth conditions for each experimental condition was used for each strain. Cultures were incubated at 30°C, with the exception of 37°C for *M. capsulatus* Bath, with shaking at 150 rpm until the early stationary phase is reached, as determined by the growth curves (OD_540_) (4).

### Adaptation of *M. album* BG8 to low pH and analysis of genome-wide mutations

A series of NMS medium formulations from pH 6.8 to 3.8 in 0.2 pH unit increments were generated using mixtures of KH_2_PO_4_, Na_2_HPO_4_, and citrate phosphate buffers with further acidification by dropwise addition of 0.1 M HCl to achieve the desired final pH prior to autoclaving. Cultures were grown on methane/air (30%/70%) and passaged a minimum of three times before moving to the next lower pH medium. Adaptation experiments were performed from 2022 to 2024. Visualization of the adapted cells was performed by TEM as described below. Genomic DNA was extracted from three replicated culture lines from parental (non-adapted) and pH 4-adapted *M. album* BG8 grown on NMS medium at pH 6.8 using the DNeasy Blood and Tissue Kit (Qiagen), cleaned using the ZymoDNA clean and concentrator kit (Zymo Research, USA), and measured by the Qubit dsDNA broad range assay (Fisher Scientific, USA). Genomes were sequenced using Ilumina Novoseq (Genome Quebec, Montreal) with 1,060× (adapted) and 1,173× coverage. The adapted genome was aligned to the parental genome using SPAdes (v4.2.0+galaxy0). Snippy (v4.6.0+galaxy0) was used to identify mutations in the consensus adapted genome.

### Transmission electron microscopy imaging

Cells (ca. 2 mL) were removed from cultures and pelleted by centrifugation (5 min at 6,000 × *g*; Eppendorf). Cell pellets were rinsed and resuspended in 500 µL phosphate buffer. Cell samples were placed on a 300-nm copper grid for 4–6 min prior to staining with 1% phosphotungstic acid. Images were captured using a Philips/FEI Transmission Electron Microscope with a Gatan Camera (Morgagni). Figure 2 was imaged with a JEOL JEM-ARM200CF S/TEM electron microscope at an accelerating voltage of 200 kV. The HAADF-STEM images were collected with the JEOL HAADF detector using the following experimental conditions: probe size, 6C; condenser lens aperture, 30 μm; scan speed, 32 μs per pixel; and camera length, 8 cm. The average diameter of S-layer proteins starting from the outer edge was measured using ImageJ (*n* = 50).

### S-layer purification

**Fig 5:**
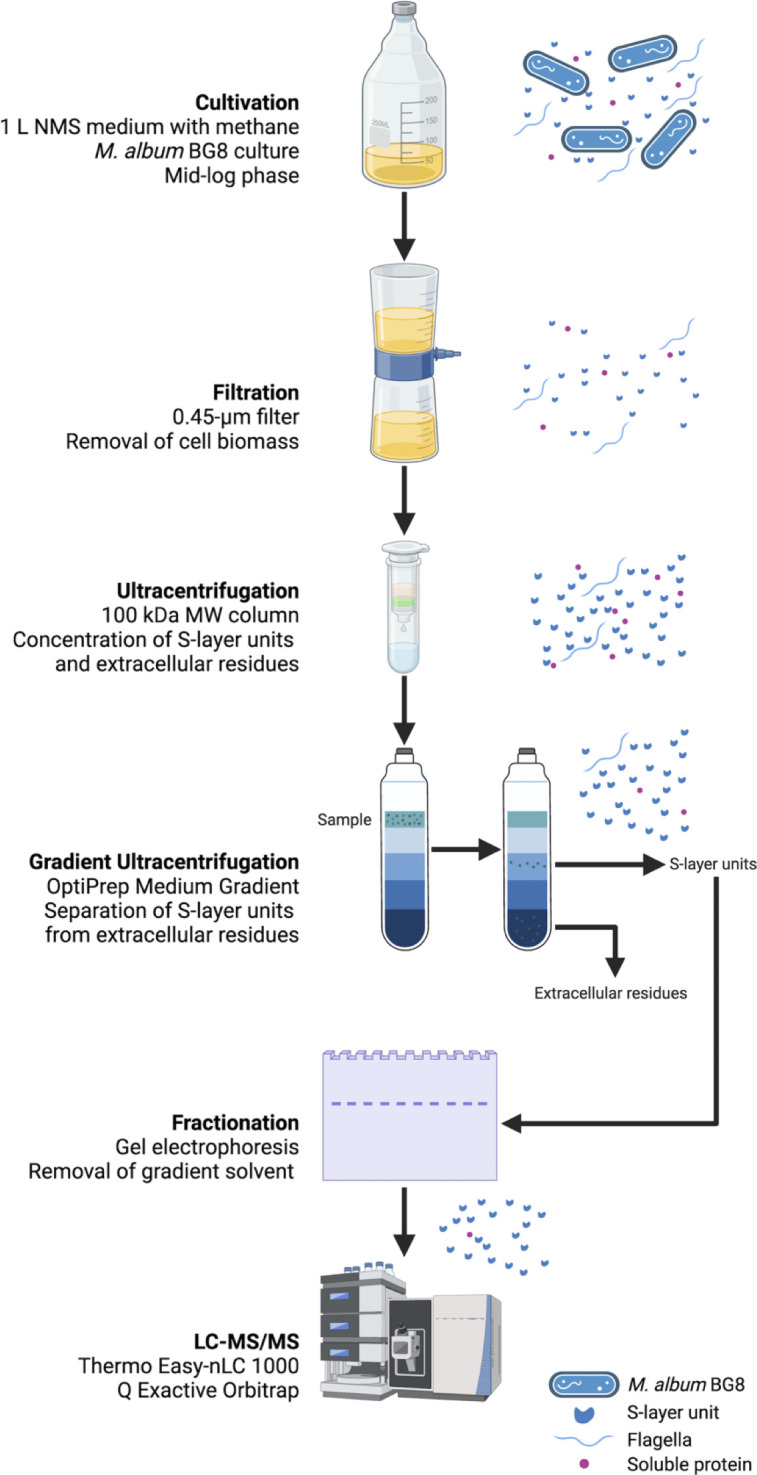
S-layer isolation and purification protocol based on density gradient centrifugation for *M. album* BG8 adapted from a protocol for OMV isolation (adapted from reference 36 with permission of the publisher).

Three technical replicates were used for purification and LC MS/MS proteomics, with each replicate deriving from 1 L of *M. album* BG8 culture grown in NMS medium with methane. The purification method is illustrated in Fig. 5 and was adapted from a protocol for isolation of outer membrane vesicles (36). For each replicate, the cultures were filtered using a 0.45-μm PES stericup (Millipore) to separate biomass from filtrate containing the S-layer proteins. The filtrate was passed through a 100 kDa MW cutoff protein concentrator (GE Vivaspin), and the retentate was collected by rinsing the filter with 1 mL phosphate buffer at pH 6.8. Retentate aliquots were spotted on NMS agar and incubated with methane to confirm the absence of viable *M. album* BG8 cells.

The crude retentate was mixed with OptiPrep (iodixanol; Sigma-Aldrich) (60%) and placed at the bottom of a sterile centrifuge tube. A discontinuous density gradient was created by layering 2 mL of diluted OptiPrep (40%, 35%, 30%, 25%, 20% using OptiPrep Diluent) as per the manufacturer’s instructions. Samples were centrifuged for 12 h at 53,000 rpm (~288,350 × *g*) at 4°C in a Ti70 Fixed rotor ultracentrifuge (Beckman Optima L-90K Ultracentrifuge); 1-mL fractions were removed by pipetting from the top of the density gradient, with a total of 18 fractions per replicate. Each fraction was measured for refractive index (Reichert AR200 Automatic Digital Refractometer) to ensure that the density gradient was maintained. Per fraction, 50 μL was denatured using 50 μL of 2× Laemmli Sample Buffer (BioRad) and incubated on a heat block (Baxter Canlab H2025-1 Dry Block Heater) at 95°C for 5 min. The prepared samples were separated on an 8% acrylamide gel and stained with colloidal Coomassie to determine the presence of proteins. Protein-containing fractions were denatured and concentrated using a protein centrifuge concentrator (Amicon) to a final volume of 50 μL. Concentrated protein samples were partially resolved through a precast 4%–20% acrylamide gradient gel (BioRad) to remove the Optiprep solvent until no residual sample was present in the wells of the gel. Protein samples were excised from the gel and submitted for LC-MS/MS analysis.

### In-gel digest of S-layer peptides

The gel bands were cut into small pieces and destained four times with 50% acetonitrile (ACN; Sigma) in 50 mM trietylammonium bicarbonate (TEAB; Sigma) at 37°C for 10 min. Destained gel pieces were washed with 100 mM TEAB at 37°C for 10 min and dehydrated by incubating them twice in 100% ACN at room temperature for 10 min to complete drying. Cysteine residues were reduced by incubating dried gel slices in 10 mM dithiothreitol (DTT; BioRad) solution in 100 mM TEAB for 45 min at 37°C. The reduction solution was replaced with 55 mM iodoacetamide (IA; Sigma) in 100 mM TEAB buffer and incubated in the dark for 1 h at 37°C to alkylate cysteine residues. The alkylation solution was replaced by 100 mM TEAB and incubated for 10 min, repeated twice. Gel pieces were then dehydrated once more in 100% ACN and dried as stated above. Gel pieces were rehydrated by adding 6 ng/μL trypsin (Promega; V5113) in 100 mM TEAB. Peptides were digested for 16 h at 37°C with shaking at 150 rpm. Tryptic peptides were retained, and in-gel-digested peptides were further extracted by incubating in 1% (vol/vol) formic acid (FA; Fisher), 2% (vol/vol) ACN in 100 mM TEAB for 1 h at 37°C. This was followed by a second 1-h extraction at 37°C using a 1:1, 1% (vol/vol) FA in 100 mM TEAB and 100% ACN extraction buffer. Digested peptide fractions were pooled and dried. Isolated peptides were re-suspended and desalted using ZipTip C18 pipette tips (ZTC18S960; Millipore), as described (37). All peptides were dried and re-suspended in 3% (vol/vol) ACN/0.1% (vol/vol) FA immediately prior to mass spectrometry analysis.

### Liquid chromatography mass spectrometry

Re-suspended tryptic peptides were analyzed using nano flow HPLC (Easy-nLC 1000, Thermo Scientific) with an EASY-Spray capillary HPLC column (PepMap RSLC C18, 75 µm × 25 cm, 100Å, 2 μm, Thermo Scientific) coupled to a Q Exactive Orbitrap mass spectrometer (Thermo Scientific). Dissolved samples were injected using an Easy-nLC 1000 system (Thermo Scientific). The column was equilibrated with 100% solvent A (0.1% [vol/vol] FA, 4% [vol/vol] ACN in water). Peptides were eluted using the following gradient of solvent B (0.1% [vol/vol] FA in 80% [vol/vol] ACN): 0%–18% B, 0–73 min; 18%–30% B, 73–101 min; 30%–46% B, 101–120 min; 46%–100% B, 120–123 min at a flow rate of 0.35 µL min^−1^ at 50°C. High-accuracy mass spectra were acquired in data-dependent acquisition mode. All precursor signals were recorded in a mass range of 300–1,700 m/z and a resolution of 35,000 at 200 m/z. The maximum accumulation time for a target value of 1 × 10^6^ was set to 120 ms. Up to 12 data-dependent MS/MS were recorded using quadrupole isolation with a window of 2 Da and higher-energy collisional dissociation fragmentation with 26% fragmentation energy. A target value of 5 × 10^4^ was set for MS/MS using a maximum injection time of 250 ms and a resolution of 17,500 at 200 m/z. Precursor signals were selected for fragmentation with charge states from +2 to +7 and a signal intensity of at least 1 × 10^4^. All precursor signals selected for MS/MS were dynamically excluded for 30 s.

All acquired mass spectrometry data were analyzed using Proteome Discoverer (ver. 2.4.1.15). Data were searched against the *M. album* BG8 proteome (UNIPROT H8GRI8_METAL; containing 3,738 protein sequences) using an automatically generated decoy database and the Proteome Discoverer processing workflow (PWF_QE_Basic_SeaquestHT) and consensus workflow (CWF_Basic) with minimal deviation. Deviations included a precursor and fragment mass tolerance of 10 ppm and 0.6 Da, respectively, trypsin digestion with two missed cleavages, variable modifications methionine oxidation (+15.995 Da), N-terminal acetylation (+42.011 Da), methionine loss (−131.040 Da), methionine loss +n-terminal acetylation (−89.030 Da), and fixed modifications carbamidomethylation (+57.021 Da).

### Identifying similar protein sequences in other methanotrophs

To determine if identified S-layer-associated proteins were unique to *M. album* BG8, each identified protein sequence was compared using BLASTp to the gammaproteobacterial methanotrophs: *Methylomonas denitrificans* FJG1 (taxon: 416); *Methylicorpusculum oleiharenae* XLMV4T (taxon: 1,338,687); *Methylotuvimicrobium buryatense* 5GB1 (taxon: 1,338,687); and *Methylococcus capsulatus* Bath (taxon: 414). (https://blast.ncbi.nlm.nih.gov/). The presence of T1SS proteins identified in the *M. album* BG8 genome (GenBank: CM001475.1) was also analyzed for their presence in the above gammaproteobacterial genomes. For query sequences with a high number of BLASTp hits, only matches with the highest percent identities to the query were reported.

To determine the presence of S-layer related proteins in gammaproteobacterial methanotrophs that were not analyzed in this experiment, the proteins of interest were screened against the entire *Methylococcaceae* group (taxid: 135,618) using BLASTp (Table S3). The S-layer protein H8GFV3 was further screened via BLASTp against all Gammaproteobacteria (taxid: 1236) (Table S4).

### RNA extraction and RNA-seq analysis

Total RNA was extracted from triplicate late-log phase cultures of the low-pH-adapted *M. album* BG8 grown in NMS medium at pH 6.8 (Fig. S1) using the QIAWave RNA Mini Kit (Qiagen) following the manufacturer’s protocol. The extracted RNA was quantified using the Qubit RNA high-sensitivity assay (Fisher Scientific, USA) and quality-controlled using the Agilent 2100 Bioanalyzer. RNA libraries were prepared by Genome Quebec (Montreal, Canada) using a bacterial rRNA-depleted RNA-seq library preparation workflow, followed by sequencing on an Illumina NovaSeq (PE100, ~25 M reads per sample, ±5 M). Raw paired-end RNA-seq reads were analyzed using the Galaxy web platform (usegalaxy.eu; version 25.0). First, reads were quality-filtered using Trimmomatic (v0.39 + galaxy2) with default parameters. High-quality reads were mapped to the low-pH adapted *M. album* BG8_1aa genome using HISAT2 (v2.2.1+galaxy0). The number of reads mapped to each gene was calculated by HTseq-count (v2.0.9+galaxy0), and the output was used to calculate normalized transcript abundances in transcripts per million (TPM). Statistically validated TPM values for the same genes from the parental *M. album* BG8 strain grown in NMS medium with methane were used from a prior RNA-seq experiment with triplicate samples (5).

## Data Availability

All raw proteomics files were submitted to the PRoteomics IDEntification Database under submission access: https://www.ebi.ac.uk/pride/archive/projects/PXD050176. The genome and RNAseq data for low pH-adapted M. album BG8 grown in NMS at pH 6.8 can be found under GenBank accession number PRJNA1402466.Transcriptome data for parental M. album BG8 grown in NMS at pH 6.8 can be found under accession number PRJNA698057. Further information on the genome mutations and calculations of RNA-seq TPM values for the low pH-adapted strain are in the Data Set S1.

## SUPPLEMENTAL MATERIAL

Data Set S1 aem.00607-26-s0001.xlsx Genome and RNAseq data analysis.

Supplemental material aem.00607-26-s0002.docx Fig. S1; Tables S1 to S4.

ASM does not own the copyrights to Supplemental Material that may be linked to, or accessed through, an article. The authors have granted ASM a non-exclusive, world-wide license to publish the Supplemental Material files. Please contact the corresponding author directly for reuse.

## References

[1] Khmelenina VN, But SY, Rozova ON, Trotsenko YA. 2019. Metabolic features of aerobic methanotrophs: news and views. Curr Issues Mol Biol 33:85–100. doi:10.21775/cimb.033.085 31166186

[2] Kang NK, Chau THT, Lee EY. 2024. Engineered methane biocatalysis: strategies to assimilate methane for chemical production. Curr Opin Biotechnol 85:103031. doi:10.1016/j.copbio.2023.103031 38101295

[3] Sauvageau D, Stein LY, Arenas E, Das S, Iacobelli M, Lawley M, Lazic M, Rondón FL, Weiblen C. 2024. Industrializing methanotrophs and other methylotrophic bacteria: from bioengineering to product recovery. Curr Opin Biotechnol 88:103167. doi:10.1016/j.copbio.2024.103167 38901110

[4] Tays C, Guarnieri MT, Sauvageau D, Stein LY. 2018. Combined effects of carbon and nitrogen source to optimize growth of proteobacterial methanotrophs. Front Microbiol 9:2239. doi:10.3389/fmicb.2018.02239 30319568 PMC6167414

[5] Sugden S, Lazic M, Sauvageau D, Stein LY. 2021. Transcriptomic and metabolomic responses to carbon and nitrogen sources in Methylomicrobium album BG8. Appl Environ Microbiol 87:e0038521. doi:10.1128/AEM.00385-21 33893121 PMC8316095

[6] Villada JC, Duran MF, Lim CK, Stein LY, Lee PKH. 2022. Integrative genome-scale metabolic modeling reveals versatile metabolic strategies for methane utilization in Methylomicrobium album BG8. mSystems 7:e0007322. doi:10.1128/msystems.00073-22 35258342 PMC9040813

[7] Pourhassan N. Z, Smits SHJ, Ahn JH, Schmitt L. 2021. Biotechnological applications of type 1 secretion systems. Biotechnol Adv 53:107864. doi:10.1016/j.biotechadv.2021.107864 34767962

[8] Khmelenina VN, Suzina NE, Trotsenko IA. 2013. Surface layers of methanotrophic bacteria. Mikrobiologiia 82:515–527. 25509389

[9] Trotsenko YA, Khmelenina VN. 2002. The biology and osmoadaptation of haloalkaliphilic methanotrophs. Microbiology (Reading, Engl) 71:123–132. doi:10.1023/A:1015183832622 12024811

[10] Shchukin VN, Khmelenina VN, Eshinimaev BT, Suzina NE, Trotsenko IA. 2011. Primary characterization of dominant cell surface proteins of halotolerant methanotroph Methylomicrobium alcaliphilum 20Z. Mikrobiologiia 80:595–605. 22168002

[11] Khmelenina VN, Shchukin VN, Reshetnikov AS, Mustakhimov II, Suzina NE, Eshinimaev BTs, Trotsenko YuA. 2010. Structural and functional features of methanotrophs from hypersaline and alkaline lakes. Microbiology (Reading, Engl) 79:472–482. doi:10.1134/S0026261710040090

[12] Hamilton R, Gebbie W, Bowman C, Mantanona A, Kalyuzhnaya MG. 2025. Microbial hauberks: composition and function of surface layer proteins in gammaproteobacterial methanotrophs. Appl Environ Microbiol 91:e0136424. doi:10.1128/aem.01364-24 39745414 PMC11784148

[13] Jeffries P, Wilkinson JF. 1978. Electron microscopy of the cell wall complex of Methylomonas albus. Arch Microbiol 119:227–229. doi:10.1007/BF00964278

[14] Kim HG, Han GH, Eom CY, Kim SW. 2008. Isolation and taxonomic characterization of a novel type I methanotrophic bacterium. J Microbiol 46:45–50. doi:10.1007/s12275-008-0017-2 18337692

[15] Bharat TAM, Kureisaite-Ciziene D, Hardy GG, Yu EW, Devant JM, Hagen WJH, Brun YV, Briggs JAG, Löwe J. 2017. Structure of the hexagonal surface layer on Caulobacter crescentus cells. Nat Microbiol 2:17059. doi:10.1038/nmicrobiol.2017.59 28418382 PMC5699643

[16] Herrmann J, Jabbarpour F, Bargar PG, Nomellini JF, Li PN, Lane TJ, Weiss TM, Smit J, Shapiro L, Wakatsuki S. 2017. Environmental calcium controls alternate physical states of the Caulobacter surface layer. Biophys J 112:1841–1851. doi:10.1016/j.bpj.2017.04.003 28494955 PMC5425405

[17] Ford MJ, Nomellini JF, Smit J. 2007. S-layer anchoring and localization of an S-layer-associated protease in Caulobacter crescentus. J Bacteriol 189:2226–2237. doi:10.1128/JB.01690-06 17209028 PMC1899406

[18] Bao K, Claesson R, Gehrig P, Grossmann J, Oscarsson J, Belibasakis GN. 2022. Proteomic characterization of the oral pathogen Filifactor alocis reveals key inter-protein interactions of its RTX toxin: FtxA. Pathogens 11:590. doi:10.3390/pathogens11050590 35631111 PMC9145396

[19] Linhartová I, Bumba L, Mašín J, Basler M, Osička R, Kamanová J, Procházková K, Adkins I, Hejnová-Holubová J, Sadílková L, Morová J, Sebo P. 2010. RTX proteins: a highly diverse family secreted by a common mechanism. FEMS Microbiol Rev 34:1076–1112. doi:10.1111/j.1574-6976.2010.00231.x 20528947 PMC3034196

[20] Bumba L, Masin J, Macek P, Wald T, Motlova L, Bibova I, Klimova N, Bednarova L, Veverka V, Kachala M, Svergun DI, Barinka C, Sebo P. 2016. Calcium-driven folding of RTX domain β-rolls ratchets translocation of RTX proteins through type I secretion ducts. Mol Cell 62:47–62. doi:10.1016/j.molcel.2016.03.018 27058787

[21] Samsudin F, Ortiz-Suarez ML, Piggot TJ, Bond PJ, Khalid S. 2016. OmpA: a flexible clamp for bacterial cell wall attachment. Structure 24:2227–2235. doi:10.1016/j.str.2016.10.009 27866852

[22] Whittenbury R, Phillips KC, Wilkinson JF. 1970. Enrichment, isolation and some properties of methane-utilizing bacteria. J Gen Microbiol 61:205–218. doi:10.1099/00221287-61-2-205 5476891

[23] von Kügelgen A, van Dorst S, Alva V, Bharat TAM. 2022. A multidomain connector links the outer membrane and cell wall in phylogenetically deep-branching bacteria. Proc Natl Acad Sci USA 119. doi:10.1073/pnas.2203156119 PMC9388160 35943982

[24] Spitz O, Erenburg IN, Beer T, Kanonenberg K, Holland IB, Schmitt L. 2019. Type I secretion systems-one mechanism for all. Microbiol Spectr 7. doi:10.1128/microbiolspec.PSIB-0003-2018 PMC11588160 30848237

[25] Fagan RP, Fairweather NF. 2014. Biogenesis and functions of bacterial S-layers. Nat Rev Microbiol 12:211–222. doi:10.1038/nrmicro3213 24509785

[26] Sander C, Schneider R. 1991. Database of homology-derived protein structures and the structural meaning of sequence alignment. Proteins 9:56–68. doi:10.1002/prot.340090107 2017436

[27] González JM, Mayer F, Moran MA, Hodson RE, Whitman WB. 1997. Microbulbifer hydrolyticus gen. nov., sp. nov., and Marinobacterium georgiense gen. nov., sp. nov., two marine bacteria from a lignin-rich pulp mill waste enrichment community. Int J Syst Evol Microbiol 47:369–376. doi:10.1099/00207713-47-2-369 9103623

[28] Hamilton R, Gebbie W, Bowman C, Mantanona A, Kalyuzhnaya MG. 2024. Microbial hauberks: composition and function of surface layer proteins in gammaproteobacterial methanotrophs. bioRxiv. doi:10.1101/2024.07.09.602694 PMC11784148 39745414

[29] Johnson KA, Ve T, Larsen Ø, Pedersen RB, Lillehaug JR, Jensen HB, Helland R, Karlsen OA. 2014. CorA is a copper repressible surface-associated copper(I)-binding protein produced in Methylomicrobium album BG8. PLoS One 9:e87750. doi:10.1371/journal.pone.0087750 24498370 PMC3912023

[30] von Kügelgen A, Tang H, Hardy GG, Kureisaite-Ciziene D, Brun YV, Stansfeld PJ, Robinson CV, Bharat TAM. 2020. In situ structure of an intact lipopolysaccharide-bound bacterial surface layer. Cell 180:348–358. doi:10.1016/j.cell.2019.12.006 31883796 PMC6978808

[31] Saidi-Mehrabad A, Kits DK, Kim J-J, Tamas I, Schumann P, Khadka R, Strilets T, Smirnova AV, Rijpstra WIC, Sinninghe Damsté JS, Dunfield PF. 2020. Methylicorpusculum oleiharenae gen. nov., sp. nov., an aerobic methanotroph isolated from an oil sands tailings pond. Int J Syst Evol Microbiol 70:2499–2508. doi:10.1099/ijsem.0.004064 32559826

[32] Pham DN, Nguyen AD, Lee EY. 2022. Outlook on engineering methylotrophs for one-carbon-based industrial biotechnology. Chem Eng J 449:137769. doi:10.1016/j.cej.2022.137769

[33] Kalyuzhnaya M, Demidenko O, Collins D. 2018. Compositions and methods using methanotrophic s-layer proteins for expression of heterologous proteins. USA.

[34] Sára M, Sleytr UB. 2000. S-Layer proteins. J Bacteriol 182:859–868. doi:10.1128/JB.182.4.859-868.2000 10648507 PMC94357

[35] Puri AW, Owen S, Chu F, Chavkin T, Beck DAC, Kalyuzhnaya MG, Lidstrom ME. 2015. Genetic tools for the industrially promising methanotroph Methylomicrobium buryatense. Appl Environ Microbiol 81:1775–1781. doi:10.1128/AEM.03795-14 25548049 PMC4325140

[36] Klimentová J, Stulík J. 2015. Methods of isolation and purification of outer membrane vesicles from gram-negative bacteria. Microbiol Res 170:1–9. doi:10.1016/j.micres.2014.09.006 25458555

[37] Uhrig RG, Schläpfer P, Roschitzki B, Hirsch-Hoffmann M, Gruissem W. 2019. Diurnal changes in concerted plant protein phosphorylation and acetylation in Arabidopsis organs and seedlings. Plant J 99:176–194. doi:10.1111/tpj.14315 30920011

